# P-580. A randomized controlled trial evaluating virologic and renal outcomes after switching from TDF/FTC/EFV to TDF/3TC/DTG (TLD) versus DTG+3TC in virologically suppressed Thai PWH – a pilot study

**DOI:** 10.1093/ofid/ofae631.778

**Published:** 2025-01-29

**Authors:** Samadhi Patamatamkul, Sathaporn Kanogtorn, Opass Putcharoen

**Affiliations:** Department of Medicine, Faculty of Medicine, Mahasarakham University, Maha Sarakham, Maha Sarakham, Thailand; Department of Medicine, Faculty of Medicine, Mahasarakham University, Maha Sarakham, Maha Sarakham, Thailand; Division of Infectious Disease, Department of Medicine, Faculty of Medicine, Chulalongkorn University, Krungthep, Krung Thep, Thailand

## Abstract

**Background:**

TDF/3TC/DTG (TLD) has been adopted as the first line of therapy for all PWH according to the WHO 2019 and Thai HIV guidelines, hence, PWH in Thailand on TDF/FTC/EFV are opted for a switch to TLD. However, switching to DTG+3TC is not inferior to TDF-based ART in terms of efficacy, but with more favorable renal safety. In this study, we compared estimated glomerular filtration rates (eGFR), virological and immunological response, and metabolic parameters among PWH currently receiving TDF+FTC or 3TC+EFV who are switched to TLD versus DTG+3TC.

**Figure 1.**
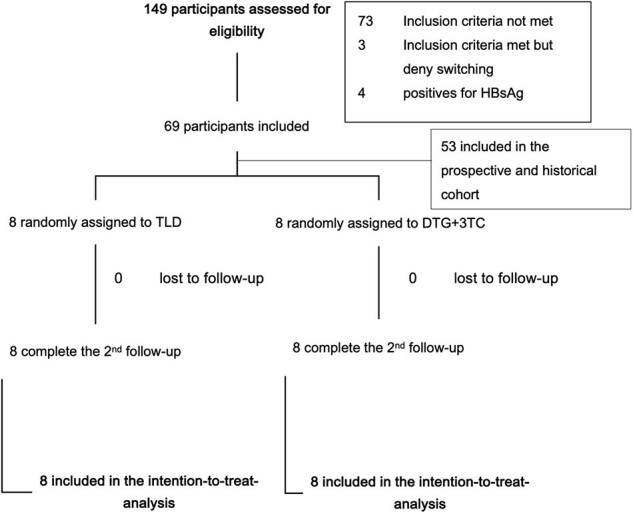
Flow diagram of eligible cases

**Methods:**

We enrolled virologically suppressed PWH age ≥18 years currently on TDF+FTC or 3TC+EFV and randomly switched to TLD or DTG+3TC at 2 tertiary care hospitals. The primary outcome was a change of eGFR calculated by cystatin C at 24 weeks. Secondary outcomes were changes in eGFR calculated by creatinine, LDL, body weight, and BMI at 24 weeks.

**Figure 2.**
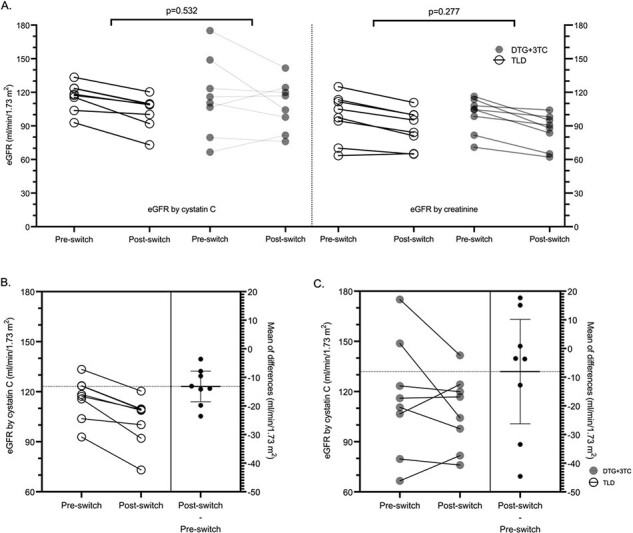
Comparison of eGFR changes calculated by cystatin C (left Y axis) and creatinine (right Y axis) from baseline (pre-switch) to the 2nd follow-up (post-switch) among participants who were switched to TLD versus DTG+3TC (A). Changes of eGFR calculated by cystatin C from pre-switch to post-switch in the TLD group (B) and DTG+3TC group (C). The differences in change for each group were shown in the scatter dot plot on the right column within each figure. The error bar represents a 95% confidence interval of the mean change, while the thick line in the middle of the bar represents the mean.

**Results:**

There were 16 participants, with 8 assigned to the TLD and 8 to the DTG+3TC group (**Figure 1**). There were 69% male with a mean age of 41 years (**Table 1**). The mean time from HIV diagnosis to switching was 7.1 years. The decrement of the eGFR by cystatin C and creatinine was higher in the TLD than in the DTG+3TC group: mean difference 5.15 ml/min/1.73 m^2^ (95% CI -12.06 to 22.35) (p=0.532) and 4.01 ml/min/1.73 m^2^ (95% CI -3.58 to 11.59) (p=0.277), respectively (**Figure 2A**). However, within each group, there was a significant reduction of eGFR by cystatin C in the TLD group: mean changes -13.19 (95% CI -18.53 to -7.84) (p< 0.001) (**Figure 2B**). There was no significant reduction of eGFR by cystatin C in the DTG+3TC group: mean changes -8.04 (95% CI -26.24 to 10.16) (p=0.331) (**Figure 2C**). In the sensitivity analysis excluding outliers, there was a significant reduction of eGFR by cystatin C after switching to the TLD than to the DTG+3TC: mean difference of 15.47 (95% CI 4.81 – 26.13) (p=0.008). All participants in both groups achieved virological suppression. There was insignificant BMI, LDL, and CD4 change between the two groups (**Figure 3**).

**Figure 3.**
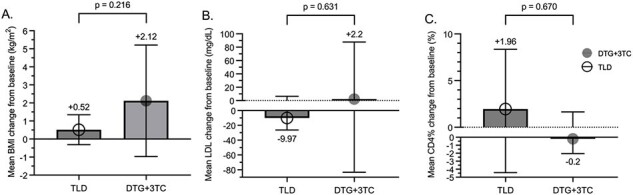
Comparison of mean changes for (A) BMI; (B) LDL; and (C) CD4 percentage (CD4%) from baseline to the 2nd follow-up after switching between the two groups. The error bar represents 95% confidence interval of mean changes calculated from paired T-tests within each group.

**Conclusion:**

There was a trend toward a higher reduction of eGFR by cystatin C among PWH who were switched to TLD than to the DTG+3TC. Changes in the LDL and BMI were comparable. Dual therapy with DTG+3TC may be a preferred option as a switching therapy over TLD in selected cases with renal safety concerns.

**Table 1.**
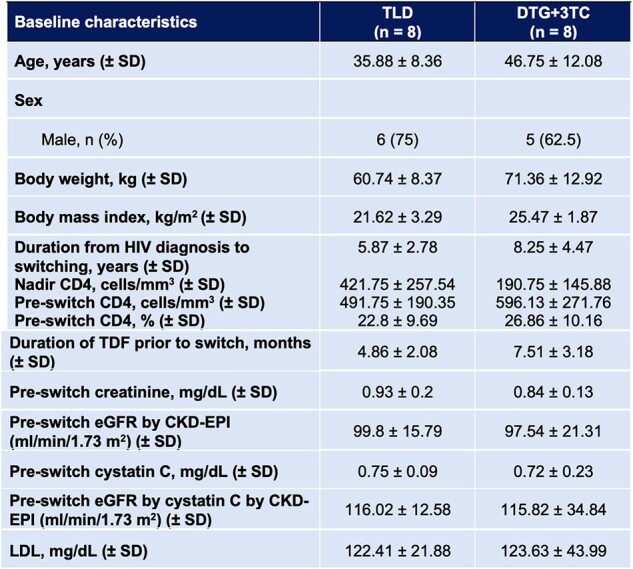
Baseline characteristics of all participants.

**Disclosures:**

**All Authors**: No reported disclosures

